# Socioeconomic inequalities in the prevalence of chronic non-communicable disease risk factors in the DIMAMO health and demographic surveillance system

**DOI:** 10.3389/fpubh.2026.1756409

**Published:** 2026-02-16

**Authors:** Khuliso Goodman Ravhuhali, Cairo Bruce Ntimana, Eric Maimela

**Affiliations:** 1 Department of Public Health, University of Limpopo, Polokwane, South Africa; 2 National Health Laboratory Service, Johannesburg, South Africa; 3 DIMAMO Population Health Research Centre, University of Limpopo, Polokwane, South Africa; 4 Department of Public Health, Faculty of Medicine and Health Sciences, Walter Sisulu University, Umtata, Eastern Cape, South Africa

**Keywords:** chronic disease, DIMAMO HDSS, non-communicable diseases, risk factors, socioeconomic inequality, South Africa

## Abstract

**Background:**

Socioeconomic inequalities play a crucial role in shaping the burden of non-communicable diseases (NCDs) and associated risk factors. This study examined socioeconomic disparities in the prevalence of chronic NCD risk factors among adults in the DIMAMO Health and Demographic Surveillance Site (HDSS) in Limpopo Province, South Africa.

**Methods:**

A cross-sectional study was conducted among adults aged 18 years and above. Data on socioeconomic status (SES) and NCD risk factors such as smoking, alcohol consumption, high salt intake, low fruit and vegetable intake, and physical inactivity were collected using an adapted WHO STEPS questionnaire. Socioeconomic status (SES) was assessed through household assets. A wealth index was constructed using principal component analysis to classify participants into SES quintiles. Concentration indices (CIs) and concentration curves were computed to assess the magnitude and direction of SES-related inequalities. Inequality decomposition analysis was performed to quantify the contribution of SES to each risk factor's inequality.

**Results:**

Participants were predominantly female (63%) and younger than 50 years (58%), with the majority unemployed (60.3%) and possessing secondary-level education (63.1%). Low fruit and vegetable intake (88.7%) emerged as the most prevalent NCD risk factor, followed by alcohol consumption (35.2%) and hypertension (22.6%). Diabetes prevalence was significantly higher among individuals in higher SES groups (*p* = 0.0071), while smoking (*CI* = −0.149, *p* = 0.024), physical inactivity (*CI* = −0.159, *p* = 0.009), and low fruit and vegetable intake (*CI* = −0.021, *p* = 0.047) were more concentrated among the poor. In contrast, diabetes (*CI* = 0.336, *p* = 0.001) and alcohol use (*CI* = 0.090, *p* = 0.036) were significantly more prevalent among the wealthy. Decomposition analysis showed that socioeconomic status accounted for nearly all observed inequalities, indicating that wealth disparities remain the dominant driver of NCD risk distribution in this population.

**Conclusion:**

The findings of the current study reveal a dual burden of NCD risk factors across socioeconomic groups; behavioral risks are concentrated among the poor, while metabolic conditions such as diabetes are more common among the affluent. Addressing these disparities requires context-specific interventions that target both behavioral and metabolic risk factors across all socioeconomic strata.

## 1 Introduction

South Africa as a country has been experiencing a quadruple burden of disease, and about mortality, non-communicable diseases (NCDs) have been leading at 43% followed by Human Immunodeficiency Virus/Acquired Immunodeficiency Syndrome (HIV/AIDS) and Tuberculosis (TB) at 34%; other communicable diseases (and perinatal conditions, maternal causes, and nutritional deficiencies) at 14% and injuries at 10% (1). According to the World Health Organization (WHO) estimates from 2016, NCDs accounted for 71% of all-cause mortality globally. More recent WHO data indicate that by 2021, this had risen to approximately 75%, with 18 million NCD deaths occurring before the age of 70 (2–4). Over the last two decades, the burden of NCDs has increased in Sub-Saharan Africa (SSA) owing to rising rates of cardiovascular risk factors such as unhealthy diets, lack of physical activity, hypertension, obesity, diabetes, dyslipidaemia, and air pollution (5). It has been estimated that almost 60% of all deaths in South Africa were due to NCDs in 2018 (6).

Inequality in health refers to disparities in health status or access to health services between populations, with one group being better off than the other (7). Social determinants such as socioeconomic status, education level, and race or ethnicity are key risk factors of NCDs that influence a person's access to care (8). People from minority and underprivileged populations may have worse results due to a lack of access and a delay in accessing care (9). One of the leading public health problems worldwide is the disparity in health between demographic classes (10). A person's socioeconomic status results from several characteristics and variables, including their education, income, employment status, location of residence, housing, the characteristics of their neighborhoods, their gender, and their ethnicity (11). Depending on their influence over lifestyle choices, health expectations, health-seeking behaviors, and access to healthcare (12). These factors can affect the distribution of health outcomes within and between societies and result in unjust and avoidable disparities when it comes to health outcomes (13). Socioeconomic status is recognized as a key contributor to the development of NCDs, not only in South Africa but globally. In high-income nations, social disparities and their association with the distribution and treatment of NCDs and associated risk factors have been thoroughly documented (14).

The growing prevalence of NCDs in South Africa has been linked to four lifestyle risk factors: poor diet, physical inactivity, tobacco use, and excessive alcohol consumption (15, 16). A noteworthy body of research links socioeconomic disadvantage, psychosocial stress, and risk behaviors for NCDs. This study aimed to investigate the socioeconomic inequalities in the prevalence of chronic NCDs (hypertension and diabetes) risk factors among adults residing in the Dikgale, Mamabolo and Mothiba (DIMAMO) Health and Demographic Surveillance Site (HDSS) in Limpopo Province, South Africa.

## 2 Methods

### 2.1 Study design and setting

A cross-sectional study was conducted at the DIMAMO Health and Demographic Surveillance Site (HDSS) in the Capricorn District of Limpopo Province, South Africa. The DIMAMO HDSS is divided into three functional community areas (FCAs) and is composed of 51 rural and semi-rural villages (17). The residents of these FCAs are mainly Northern Sotho-speaking. The site is served by eleven Primary Health Care clinics, with one tertiary hospital approximately 10–30 km from the DIMAMO HDSS site (17, 18).

### 2.2 Study population and sampling

The study targeted adults aged 18 years and older attending primary healthcare facilities within the DIMAMO HDSS. A simple random sampling method was used to select the participants. The sample size was calculated based on a national prevalence estimate of chronic NCDs (51.8%), with a 95% confidence interval and 5% precision, resulting in a minimum sample of 407. After accounting for a 10% non-response rate, the final sample size was 448, with 398 participants completing the survey (response rate: 89%).

### 2.3 Data collection

Data were collected using a structured, interviewer-administered questionnaire adapted from the World Health Organization (WHO) STEPwise approach to NCD surveillance (STEPS) and the WHO Study on Global Aging and Adult Health (SAGE) tools (19). The questionnaire included sections on sociodemographic characteristics, lifestyle and behavioral risk factors (tobacco use, alcohol consumption, diet, and physical activity), and self-reported diagnoses of hypertension and diabetes.

Socioeconomic data were collected through questions on household characteristics, including ownership of durable assets (e.g., cars, refrigerators, televisions), housing materials, main cooking fuel, access to electricity, water source, type of sanitation, and ownership of livestock. Data collection was conducted by trained fieldworkers fluent in Northern Sotho and English. Daily supervision and quality checks were carried out to ensure completeness and accuracy.

### 2.4 Measurement of variables

#### 2.4.1 Socioeconomic status (SES)

A wealth index (WI) was constructed as a proxy measure for SES using principal component analysis (PCA), a widely used proxy measure in settings where reliable income data are difficult to obtain. The index was based on household asset ownership, housing conditions, and access to basic services, including type of dwelling materials, source of drinking water, sanitation facilities, electricity access, cooking fuel, ownership of durable assets (e.g., refrigerator, television, motor vehicle), and livestock ownership.

Income data were excluded from the SES construction due to substantial missingness (approximately 70%), which limited its reliability for inclusion in multivariable analysis. The resulting wealth index therefore, primarily reflects long-term structural household living conditions and access to basic services, rather than individual-level income or consumption patterns. Household wealth scores were ranked and categorized into five quintiles: poorest, poorer, middle, richer, and richest.

#### 2.4.2 Non-communicable disease (NCD) risk factors

The main outcome variables were self-reported hypertension and diabetes, and behavioral risk factors including current tobacco use, harmful alcohol consumption, physical inactivity, high salt intake, and low fruit and vegetable intake. These risk factor measures were adapted from WHO STEPS-aligned questions to ensure standardization and comparability with international NCD surveillance tools. Each variable was coded as binary (1 = presence, 0 = absence).

### 2.5 Data analysis

Data were entered and cleaned in Microsoft Excel and analyzed using Stata version 19.5. Descriptive statistics were used to summarize participants' sociodemographic characteristics and the prevalence of NCD risk factors across SES quintiles. Categorical variables were compared using chi-square tests, and statistical significance was set at *p* < 0.05.

Socioeconomic inequalities in NCD risk factors were examined using concentration curves and Wagstaff normalized concentration indices (CIs), which measure the degree of inequality in binary health outcomes. The model included age, sex, education level, employment status, and socioeconomic status quintiles as explanatory variables. The method assumes a linear additive relationship between determinants and the health outcome, allowing the contribution of each determinant to overall inequality to be quantified. A positive CI indicates that the risk factor is more prevalent among higher SES groups (pro-rich), while a negative CI suggests concentration among lower SES groups (pro-poor).

## 3 Results

### 3.1 Characteristics of study participants

As indicated in Table 1, most participants were aged between 31–50 years (42.0% females; 47.3% males), followed by the 18–30 age group (40.4% females; 25.7% males). The age distribution was statistically significant (*p* = 0.006). Employment was more common among males (36.5%). Smoking was significantly more prevalent among males (41.9%) than among females (2.2%). Similarly, alcohol consumption was higher among males (52.7%) than among females (31.2%). Approximately 22.6% of participants self-reported a diagnosis of hypertension, with no significant gender difference (*p* = 0.593). A higher proportion of males (18.1%) reported diabetes compared to females (4.7%), which was statistically significant (*p* < 0.001).

**Table 1:** Characteristics of the study participants in DIMAMO HDSS (*n* = 398).

**Characteristic**	**Females (*n* = 324)**	**Males (*n* = 74)**	**Total (*n* = 398)**	***P*-value**
**Age group (Years)**				
18–30	131 (40.4)	19 (25.7)	150 (37.7)	**0.007**
31–50	136 (42.0)	31 (41.9)	167 (41.9)	
≥51	57 (16.6)	24 (32.4)	81 (20.4)	
**Level of education**				0.155
No formal education	4 (1.2)	3 (4.1)	7 (1.8)	
Primary	27 (8.3)	10 (13.5)	37 (9.3)	
Secondary	208 (64.2)	43 (58.1)	251 (63.1)	
Tertiary	85 (26.2)	18 (24.3)	103 (25.9)	
**Marital status**				
Single	241 (74.4)	35 (47.3)	276 (69.3)	**< 0.001**
Married	65 (20.1)	26 (35.1)	91 (22.9)	
Divorced	12 (3.7)	6 (8.1)	18 (4.5)	
Widowed	6 (1.8)	7 (9.5)	13 (3.3)	
**Occupation**				
Employed	74 (22.8)	27 (36.5)	101 (25.4)	**< 0.001**
Pensioner	17 (5.2)	11 (14.9)	28 (7.0)	
Student	23 (7.1)	6 (8.1)	29 (7.3)	
Unemployed	210 (64.8)	30 (40.5)	240 (60.3)	
**No of household members**				
1–2	15 (4.6)	13 (17.6)	28 (7.0)	**< 0.001**
3–4	76 (23.5)	17 (22.9)	93 (23.4)	
≥5	233 (71.9)	44 (59.5)	277 (69.6)	
**Smoking any tobacco**				
Yes	7 (2.2)	31 (41.9)	38 (9.6)	**< 0.001**
No	317 (97.8)	43 (58.1)	360 (90.4)	
**Alcohol consumption**				
Yes	101 (31.2)	39 (52.7)	140 (35.2)	**< 0.001**
No	223 (68.8)	35 (47.3)	258 (64.8)	
**Self-reported hypertension**				
Yes	70 (22.1)	18 (25.0)	88 (22.6)	0.593
No	247 (77.9)	54 (75.0)	301 (77.4)	
**Self-reported diabetes**				
Yes	15 (4.7)	13 (18.1)	28 (7.2)	**< 0.001**
No	303 (95.3)	59 (81.9)	362 (92.8)	
**Income level per month (r and s)**				
1 – 2,500	48 (51.1)	11 (45.8)	59 (14.8)	0.129
2,501 – 5,000	29 (30.8)	4 (16.7)	33 (8.3)	
5,001 – 10,000	12 (12.8)	4 (16.7)	16 (4.0)	
10,001 – 20,000	3 (3.2)	3 (12.5)	6 (1.5)	
>20,000	2 (2.1)	2 (8.3)	4 (1.0)	
**SES**				
Poorer	91 (28.1)	19 (25.7)	71 (17.9)	0.286
Poor	61 (18.8)	10 (13.5)	110 (27.6)	
Middle	74 (22.8)	13 (17.6)	87 (21.9)	
Richer	47 (14.5)	16 (21.6)	63 (15.8)	
Richest	51 (15.7)	16 (21.6)	67 (16.8)	

Bold value indicates the statistical significance at *p* < 0.05.

### 3.2 Prevalence of chronic non-communicable disease risk factors

Table 2 indicates the prevalence of chronic non-communicable disease risk factors by SES. Among the NCD risk factors assessed, hypertension was the most prevalent, reported in 88 participants overall; the difference across SES groups was not statistically significant (*p* = 0.6295). Diabetes demonstrated a significant variation by SES (*p* = 0.007), with the highest prevalence observed in the “richer” (30.3%) and “richest” (21.4%) groups. Alcohol intake was the most common behavioral risk factor, affecting 140 participants overall, with relatively uniform prevalence across SES groups and no statistically significant differences (*p* = 0.597). High salt intake was most common in the “Poorer” group (25.1%), but the variation across SES quintiles was not statistically significant (*p* = 0.166). Low fruit and vegetable intake was more prevalent in the “Poorer” group (28.9%) compared to the “Richer” (15.3%), though not statistically significant (*p* = 0.111). Physical inactivity was highest among individuals in the “Poorer” SES group (39.7%) and declined with increasing SES; however, this variation was not statistically significant (*p* = 0.064).

**Table 2:** Prevalence of chronic non-communicable disease risk factors by SES among adults aged ≥18 years.

**NCD Risk Factor**	**Poorest**	**Poorer**	**Middle**	**Richer**	**Richest**	**Total**	***P*-value**
Hypertension	26 (29.5)	12 (13.6)	17 (19.30)	16 (18.2)	17 (19.3)	88 (100)	0.629
Diabetes	5 (17.9)	1 (3.6)	5 (17.9)	6 (21.4)	11 (30.3)	28 (100)	**0.007**
Smoking	7 (18.4)	7 (18.4)	7 (18.4)	11 (28.9)	6 (15.8)	38 (100)	0.976
Alcohol intake	32 (22.9)	25 (17.9)	33 (23.6)	25 (17.9)	25 (17.9)	140 (100)	0.596
High salt intake	63 (25.1)	40 (15.9)	58 (23.1)	41 (16.3)	49 (19.5)	251 (100)	0.166
Low fruit & vegetable intake	102 (28.9)	67 (18.9)	74 (20.9)	54 (15.3)	56 (15.9)	353 (100)	0.111
Physical inactivity	29 (39.7)	14 (19.2)	10 (13.7)	8 (10.9)	12 (16.4)	73 (100)	0.064

Bold value indicates the statistical significance at *p* < 0.05.

### 3.3 Concentration indices for NCD risk factors

As shown in Table 3, diabetes had the highest positive concentration index (*CI* = 0.336, 95% CI: 0.132 to 0.539, *p* = *0.001*), indicating a significant concentration of diabetes among individuals in higher socioeconomic groups. Smoking (*CI* = −0.149, *p* = 0.024) and physical inactivity (*CI* = −0.159, *p* = 0.009) were significantly concentrated among the poor. Low fruit and vegetable intake also showed a small but significant concentration among the poor (*CI* = −0.021, *p* = 0.047). Concentration indices for hypertension (*CI* = 0.045, *p* = 0.408) and high salt intake (*CI* = 0.050, *p* = 0.024) suggest mild concentration among the rich. Alcohol use (*CI* = 0.090, *p* = 0.036) was also significantly more common among higher SES groups. Estimates for alcohol use and high salt intake exhibited wide confidence intervals, indicating imprecision and suggesting that these inequalities should be interpreted with caution.

**Table 3:** Concentration indices for NCD risk factors.

**NCD risk factor**	**CI**	**SE**	**95%CI**	***P*-value**
Hypertension	0.045	0.054	−0.061, 0.151	0.408
Diabetes	0.336	0.104	0.132, 0.539	**0.001**
Smoking	−0.149	0.066	−0.278–0.019	**0.024**
Alcohol use	0.090	0.429	−0.751, 0.931	**0.036**
Low fruit & vegetable intake	−0.021	0.010	−0.041–0.001	**0.047**
High salt intake	0.050	0.220	−0.381, 0.481	**0.024**
Physical inactivity	−0.159	0.061	−0.278–0.039	**0.009**

CI = Concentration Index; SE = Standard Error; 95% CI = 95% Confidence Interval. Bold value indicates the statistical significance at *p* < 0.05.

### 3.4 Concentration curves for selected NCD risk factors

**Figure 1:**
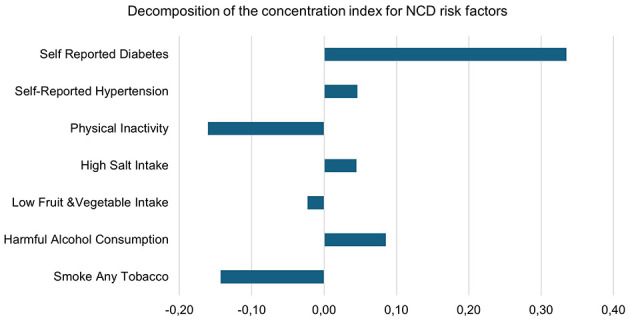
Decomposition of the concentration index for NCD risk factors.

In Figure 1, the 45-degree line represents equality, where each proportion of the population contributes equally to the cumulative burden of the risk factor. Curves above the line of equality indicate that the risk factor is more prevalent among the poor, while curves below the line suggest concentration among the wealthy. Curves for smoking, physical inactivity, and low fruit and vegetable intake lie distinctly above the line of equality, indicating that these risk factors are disproportionately concentrated among individuals in lower socioeconomic groups. The curve for diabetes lies below the line of equality, suggesting a strong concentration among wealthier individuals. Harmful alcohol intake and high salt intake have curves that track close to the line of equality, indicating a more even distribution across SES groups.

### 3.5 Decomposing Inequality for NCD risk factors

**Figure 2:**
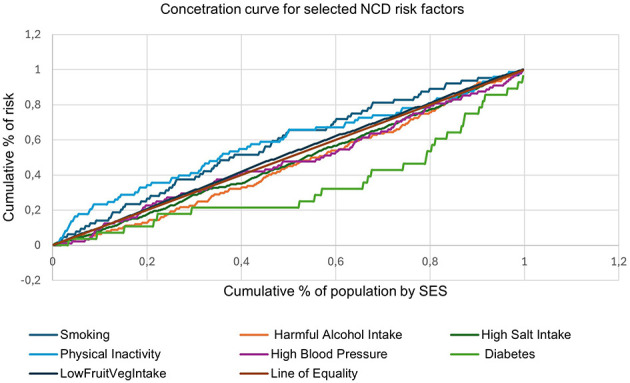
Concentration curve for selected NCD risk factors.

In Figure 2, the concentration index analysis revealed distinct socioeconomic gradients in NCD risk factors among adults in the DIMAMO HDSS population. behavioral risk factors, including smoking (*CI* = −0.14), low fruit and vegetable intake (*CI* = −0.02), and physical inactivity (*CI* = −0.16), were more prevalent among individuals in lower socioeconomic groups, indicating pro-poor inequalities. In contrast, metabolic and lifestyle-related risk factors such as alcohol consumption (*CI* = +0.09), high salt intake (*CI* = +0.05), hypertension (*CI* = +0.05), and diabetes (*CI* = +0.34) were concentrated among wealthier participants, reflecting pro-rich inequalities. This dual pattern highlights an emerging epidemiological transition in which poverty-related behavioral risks persist alongside wealth-associated metabolic diseases, underscoring the need for differentiated prevention strategies targeting both disadvantaged and affluent groups.

## 4 Discussion

This study examined socioeconomic inequalities in the prevalence of chronic NCD risk factors among adults in the DIMAMO HDSS in Limpopo Province, South Africa. The study population was relatively young, with most participants aged 18–50 years, a predominance of women, and a high unemployment rate, particularly among females. Educational attainment was mostly secondary level, and over 40% of participants belonged to lower socioeconomic quintiles. These sociodemographic attributes collectively mirror the broader socioeconomic profile of many rural South African communities, where limited employment opportunities and educational attainment contribute to poverty and restricted access to healthcare and healthy lifestyle options (18, 20).

The findings revealed disparities in the distribution of NCD risk factors across socioeconomic strata. While behavioral risk factors such as smoking, physical inactivity, and low fruit and vegetable intake were more common among individuals in lower socioeconomic groups, metabolic risk factors such as diabetes were more concentrated among wealthier participants. This pattern underscores a complex dual burden of NCD risk factors, shaped by both material deprivation and lifestyle transitions, reflecting both material deprivation and lifestyle transitions associated with urbanization and changing social determinants of health (21).

The interpretation of self-reported hypertension and diabetes warrants caution. These outcomes are likely subject to underdiagnosis, particularly among individuals in lower socioeconomic groups who may have limited access to routine screening services and healthcare services. Conversely, higher reported prevalence among wealthier participants may partly reflect better healthcare access and greater diagnostic awareness rather than true differences in underlying disease burden. Such differential reporting may partly explain the observed pro-rich concentration of diabetes in this study.

The predominance of younger adults in the sample is notable because this age group represents a critical window for NCD prevention. Similar to findings from the 2023 South African Demographic and Health Survey, younger adults often report high rates of behavioral risks, including tobacco and alcohol use, which can predispose them to metabolic disease later in life (22). The high unemployment and low income levels observed likely exacerbate vulnerability to NCD risk factors through psychosocial stress and reduced capacity to adopt healthier diets (21). Thus, the socioeconomic context of young adults in this setting may magnify their risk trajectories over time.

Gender differences were pronounced; men reported higher smoking and alcohol consumption rates, while women were more likely to be unemployed and physically inactive. These findings mirror trends reported in other African HDSS populations, where gender norms and socioeconomic constraints influence risk behaviors differently (23). In 2020, a study in rural Kenya, men exhibited significantly higher engagement in harmful alcohol and tobacco use, while women were more affected by obesity and physical inactivity (24). These parallels suggest that gendered risk patterns in this study are consistent with regional trends shaped by social roles and access to resources.

The observed concentration of smoking, physical inactivity, and poor dietary habits among individuals in lower SES groups aligns with previous studies in South Africa and other low- and middle-income countries (LMICs) that link poverty to unhealthy behavioral patterns (18, 21). Likewise, a study in Nigeria found that individuals from poorer communities exhibited higher rates of smoking and unhealthy dietary habits due to limited access to health information and economic constraints in food choices (25). Similar patterns were observed in Kenya's national STEPS survey, where unhealthy behaviors were more concentrated among rural and low-income populations (26). These consistencies reinforce the persistent influence of structural deprivation on behavioral risk exposures.

In contrast, the concentration of diabetes among higher socioeconomic groups in this study is consistent with the epidemiological transition observed in sub-Saharan Africa (5). A study of income-based inequalities across four South Asian countries found that higher income groups had a greater prevalence of NCD risk factors, including diabetes, and greater inequities in preventive care services (27). Similarly, in South Africa between 2003 and 2016, SES gradients in diabetes prevalence showed that higher SES was associated with greater prevalence of self-reported diabetes (28). In Ghana, diabetes prevalence was significantly higher among educated and urban residents compared to poorer rural populations (29). As income increases, individuals often adopt more sedentary lifestyles and consume energy-dense diets, contributing to higher rates of obesity and diabetes. This suggests that as SES improves, exposure to energy-dense diets and sedentary lifestyles may increase, leading to metabolic disorders even in contexts of overall improved living standards.

Although hypertension was the most prevalent NCD risk factor in this population, its distribution across socioeconomic quintiles did not show a statistically significant pattern. This finding is consistent with a systematic analysis of the burden of NCDs across European countries; SES inequalities in some conditions were evident, while in others they were less consistent (30). Similar results have been reported in other sub-Saharan African studies (23, 31), suggesting that hypertension may now be pervasive across all socioeconomic strata, reflecting its multifactorial aetiology involving stress, diet, genetics, and healthcare access. In rural communities, limited screening services and low awareness may suppress diagnosis among low-income groups, while in higher SES groups, better access to healthcare may lead to higher detection rates. Similar findings were reported in a national South African survey, which found no consistent socioeconomic gradient in hypertension prevalence (32). The coexistence of high hypertension prevalence across all SES levels suggests that both prevention and management strategies should target the entire population, regardless of wealth status.

The divergence in the socioeconomic patterning of behavioral and metabolic risk factors in this study supports the “inverse equity hypothesis”, which posits that new health risks and interventions initially benefit higher SES groups but later shift to lower SES groups as exposure and awareness spread. Behavioral risks such as tobacco and alcohol use may initially be more prevalent among the affluent but become increasingly concentrated among the poor as interventions target wealthier populations. Conversely, metabolic risks such as diabetes and hyperlipidaemia may initially rise among the wealthy before diffusing across socioeconomic strata as lifestyles change. Recent literature also suggests that social determinants such as material needs insecurity are strongly associated with cardiovascular risk in rural South Africa (33). A 2024 study of older adults in Mpumalanga Province found that material needs security was associated with cardiovascular risk factors (33). This underscores that beyond simple income or asset-based SES measures, deeper structural determinants matter for metabolic and behavioral risk profiles.

These findings have several important implications for NCD prevention and control. First, interventions targeting behavioral risk factors must prioritize low-income populations through community-based education, health promotion, and fiscal policies (e.g., tobacco taxation and regulation of unhealthy foods). Evidence from the South African sugar-sweetened beverage tax demonstrates that fiscal measures can reduce consumption of unhealthy products, particularly among low-income populations (34). A South African study of processed-food consumption found significant associations between processed foods and NCDs (35). Such evidence reinforces the value of population-level policies that disproportionately benefit disadvantaged groups. Second, screening programs for diabetes and hypertension should be expanded to ensure early detection across all SES groups, particularly in rural settings. Third, addressing the structural determinants of health, such as poverty, unemployment, and education, is critical to reducing NCD inequalities. The integration of social protection programs with primary health care could improve both health outcomes and equity. Moreover, strengthening surveillance systems like DIMAMO HDSS provides valuable data for monitoring health disparities over time.

### 4.1 Strengths and limitations of the study

This study is among the few to quantify socioeconomic inequalities in NCD risk factors using concentration indices in a rural South African setting. The use of standardized WHO tools and principal component analysis to derive a wealth index strengthens the reliability of the findings. However, several limitations should be acknowledged. The cross-sectional design limits causal inference between SES and NCD risk factors. Additionally, participants were recruited from primary healthcare facilities within the HDSS, which may introduce selection bias by underrepresenting individuals with limited access to health services. This could result in an underestimation of NCD risk factors, particularly among lower socioeconomic groups. Income data were excluded from the SES construction due to a high proportion of missing values (70%), which may have limited the ability of the wealth index to capture short-term monetary inequalities. Self-reported data on hypertension and diabetes may be subject to recall or diagnostic bias, particularly in poorer groups with limited healthcare access. Behavioral risk factors, including salt intake, dietary patterns, and physical activity, may be subject to recall and social desirability bias, which could vary across socioeconomic groups. The relatively wide confidence intervals observed for alcohol and salt intake inequalities may reflect limited variability or measurement error in these self-reported behaviors. Although interactions between socioeconomic status and age or sex could provide additional insights, limited sample sizes within strata constrained the feasibility of robust interaction analyses. Future studies with larger samples should explore intersectional inequalities. Despite these limitations, the study contributes important evidence on the socioeconomic distribution of NCD risk factors in underserved rural settings and highlights priority areas for intervention.

## 5 Conclusion

The study demonstrates clear socioeconomic inequalities in the prevalence of NCD risk factors in a rural South African population. behavioral risks such as smoking, poor diet, and physical inactivity are concentrated among the poor, while metabolic risks like diabetes are more common among the affluent. The findings support targeted community-based interventions to reduce behavioral risk factors among low socioeconomic groups, alongside expanded screening and early detection programmes for hypertension and diabetes across all SES strata, including higher-income populations. Addressing NCD inequalities will also require intersectoral actions integrating social protection, employment creation, and educational opportunities to address upstream determinants of health. Strengthening community-level health promotion and surveillance systems will be essential to achieving equitable NCD prevention and control in South Africa. Future studies should incorporate objective biometric measurements, such as blood pressure and blood glucose testing, to improve accuracy and reduce socioeconomic reporting bias.

## Data Availability

The raw data supporting the conclusions of this article will be made available by the authors, without undue reservation.
